# 

**Published:** 1789

**Authors:** 


### CATALOGUE OF BOOKS.

1. PLANTARUM Icones hactenus ineditæ, plerumque ad Plantas in Herbario Linnæano conservatas delineatæ. Auctore

" Lazaro compta. Erat autem Lazarus justæ staturæ, cor-

" pore decenti, moribus humanis, et ad aulæ morem ornatus.

" Inducto pallio fratris tegebat corpus fovebatque, nec mon-

" strum intus condi primo alloquio diceres. Animo ubique

" præsenti videbatur, nisi quod de fato subinde sollicitus,

" mortem fratris timebat, quod se fœtore et putredine exstin-

" guendum quoque præsagiret, hinc magis in curando fratre

" quam se laborabat."—Vide Thomæ Bartholini Hist. Anatom. rarior. Cent 1. Hist. 66.

* " In the year 1530 there was a man to be seen at Paris,

" out of whose belly another, perfect in all his members,

" except head, hanged forth as if he had been grafted there.

" The man was forty years old, and he carried the other,

" implanted or growing out of him, in his arms, with such

" admiration to the beholders, that many ran very earnestly

" to see him."—English Translation of Paré's Works by Tho. Johnson, folio, London, 1678, page 587.—See also, for another description of the same monster, Licetus de Monstris, ex recensione Blasii, 4to. Amstel. 1665, p. 83.

*Jacobo*

*Jacobo Edvardo Smith*, M. D. Societ. Reg. Lond. Uliffip. Agron. Paris. Socio, Soc. Linnæanæ Londinenfis Præfidi. Fafciculus I. Folio. Londini, 1789.

2. Hortus Kewenfis; or, A Catalogue of the Plants cultivated in the Royal Botanic Garden at Kew. By *William Aiton*, Gardener to His Majefty. In Three Volumes. 8vo. *Nicol*, London, 1789.

3. Medical Inquiries and Obfervations; to which is added an Appendix, containing Obfervations on the Duties of a Phyfician, and the Methods of improving Medicine. By *Benjamin Rufh*, M. D. Profeffor of Chemiftry in the Univerfity of Pennfylvania. The Second Edition. 8vo. *Dilly*, London, 1789.

4. A Differtation on the Procefs of Nature in the filling up of Cavities, healing of Wounds, and reftoring Parts which have been deftroyed in the human Body; which obtained the Prize Medal given by the Lyceum Medicum Londinenfe, for the Year 1789, and was ordered to be printed for the Ufe of the Society. By *James Moore*, Member of the Surgeons Company of London. 4to. *Johnfon*, London, 1789.

5. An Account of the Nature, Properties, and medicinal Ufes of the Mineral Water at

Nottington, near Weymouth, Dorfet. By *J. Crane*, Phyfician at Dorchefter. With a View of its Well in its prefent State. 8vo. *Newberry*, London, 1789.

6. Speculations on the Mode and Appearances of Impregnation in the human Female; with an Examination of the prefent Theories on Generation. By a Phyfician. 8vo. *Elliot*, Edinburgh, 1789.

7. An Account of the various Syftems of Medicine from the Days of Hippocrates to the prefent Time; collected from the beft Latin, French, and Englifh Authors, particularly from the Works of John Browne, M. D. By *Francis Carter*, M. D. 2 Vols. 8vo. *Murray*, London, 1789.

8. A Treatife on Fevers, wherein their Caufes are exhibited in a new Point of View; by a proper Attention to which, their Contagion (accruing from a contaminated Air) will be prevented; and a Variety of Cafes, as putrid Sore Throats, Inflammations, Fluxes, Influenzas, Confumptions, as well as low nervous Fevers that terribly affect the Spirits, may be cured with Eafe. 8vo. *Scatcherd*, London, 1788.

9. A Treatife upon the Herb Tobacco, pointing out its deleterious, pernicious Quality, and its fatal Effects upon the human Constitution, by the great Variety of Disorders it occasions; not only affecting three of the five Senses to a great Degree, but impairing the Faculties of the Mind, and even frequently causing premature Death. By a Gentleman of the University of Cambridge. 8vo. *Stalker*, London, 1789.

10. A new compendious System on several Diseases incident to Cattle, wherein the Disorders are orderly described, and the Symptoms of each Disease obviously laid down; together with a complete Number of Medicines for every Stage and Symptom thereof. There is also annexed an Essay on the Diseases incident to Calves, and their curative Indications. In the Course of this Work will be found several Observations on the Diseases peculiar to Horses, and their proper Method of Treatment. By *Thomas Topham*. 8vo. *Scatcherd*, London, 1788.

11. A Tale of Truth addressed to Arthritics; containing a secure, cheap, and certain Remedy for the Gout. 8vo. *Kearsley*, London, 1789.

12. Medical Essays *. 1. An Essay on the

* See Vol. II. page 222; and Vol. V. page 105.

Principles and Manners of the medical Profession; 2. An Inquiry into the Merits of Solvents for the Stone. With Additions. 8vo. *Dodsley*, London, 1789.

13. An Essay on the erysipelatous Sore Throat: to which is added an Account of a Case of Hemiplegia. By *Thomas Reeve*, Surgeon, Botesdale. 8vo. *Richardson*, London, 1789.

14. *Antonii Laurentii de Jussieu*, Regi a Consiliis et Secretis, Doctoris Medici Parisiensis, Regiæ Scientiarum Academiæ Regiæque Societatis Medicæ Parisiensis, necnon Academiarum Upsal. Matrit. Lugd. Socii, et in Horto Regio Paris. Botanices Professoris, Genera Plantarum secundum Ordines naturales disposita, juxta Methodum in Horto Regio Parisiensi exaratam, Anno 1774. 8vo. Parisiis, 1789.

15. *Georg. Gustav. Dētharding*, Medicinæ et Chirurgiæ Doctoris, et Pratici Rostochiensis, Commentatio Chirurgico-obstetrica de Utero inverso; simul Prælectiones per semestre hybernum habendas indicit. 8vo. Rostoch, 1788.

16. Commentatio de Generatione crustæ sic dictæ inflammatoriæ, secundum mentem Hewsoni. Auctore *Geo. Gust. Dētharding*, Med. et Chir. Doct. 8vo. Jena, 1788.

17. Differ-

17. Diſſertatio phyſico-medica de Magnetiſmo, et minerali et animali. Auctore *Joanne Andr. Franc. Kumpel.* 4to. Jena, 1788.

18. Diſſertatio medica de Natatione frigidâ magno Sanitatis præſidio. Auctore *Valer. Guielm. Neubeck.* 4to. Jena, 1788.

19. Regii Inſtituti veterinarii Hafnienſis brevem hiſtoriam ſcripſit *F. C. Abilgaard*, Med. Doctor et Artis veterinariæ Profeſſor. 8vo. Havniæ, 1788.

20. Diſſertatio Inauguralis medica ſiſtens Toxicologiam veterum, Plantas venenatas deſcribentem veteribus cognitas. Auctore *J. E. Ferdinand. Schulze*, Halenſi. 4to. Halæ, 1788.

21. Diſputatio medica "utrum ex recentio" ris chemiæ detectis, vero ſimilior aſſignari "queat animalis caloris origo?" Auctore *Jacobo Joſepho Audirac*, Cameracenſi, Equite, Doctore medico Monſpelienſe, Societatis Regiæ Scientiarum Monſpel. Socio, nec non ſaluberrimæ Facultatis Medicinæ Pariſienſis Baccalaureo. 4to. Pariſiis, 1788.

22. *Henrici Friderici Link*, Hildeſienſis, Commentatio de Analyſi Urinæ et origine Calculi; in concertatione Civium Academiæ Georgiæ Auguſtæ 4 Junii 1788, præmio a Rege M.

Britanniæ

Britanniæ Aug. conſtituto ab medicorum ordine ornata. 4to. Gottingæ, 1788.

23. Tentamen Phyſiologicum de Vaſis Lymphaticis. Auctore *Renato Nicolao du Friche des Genettes*, Sagienſi in Neuſtriâ. 8vo. Monſpelii, 1789.

24. Diſſertatio Inauguralis medico-obſtetricia ſiſtens Comparationem inter verſionis negotium et Operationem inſtrumentalem. Auctore *Aug. Ludov. Guilielmo Mitkoff*, Sverino-Megapolitano. 8vo. Gottingæ, 1789.

25. De Laude Magnetiſmi ſic dicti Animalis ambigua; Oratio habita ſub auſpiciis Prorectoratus in Georgia Auguſta d. 2 Julii, A. 1789, ſuſcepti ab *J. Andrea Murray*. 4to. Gottingæ, 1789.

26. *Georgii Chriſtophori Siebold*, Wirceburgenſis, Commentatio de effectibus Opii in corpus animale ſanum maxime reſpectu habito ad ejus analogiam cum vino; in concertatione Civium Academiæ Georgiæ Auguſtæ 4 Junii, 1789, præmio a Rege M. Britanniæ Aug. conſtituto ab medicorum ordine ornata. 4to. Gottingæ, 1789.

27. Diſſertatio Inauguralis Medica de Febris Phthiſicorum Natura et Curatione; Auctore

*Anton.*

*Anton. Frider. Guilielm. Hartel*, Hildesiensi. 8vo. Gottingæ, 1788.

28. Het Geneeskundig Journael van London, voor het jaer 1786; uyt het Engelsch vertaeld, door *F. A. Van Zandycke*, M. D. 8vo. Brugge, 1788.

29. Anatomische Tabellen für Lehrlinge der Anatomie, &c. *i. e.* Anatomical Tables for Students of Anatomy: by *J. A. Kulmus*; improved, with the Addition of twenty-seven Plates, by *K. G. Kuhn*, M. D. and Professor Extraordinary of Anatomy at Leipsick. 4to. Leipsick, 1789.

30. Memoires de l'Academie Royale des Sciences. Années 1786-87. 4to. Turin, 1788.

31. Notice des Insectes de la France, reputés venimeux, tirée des Ecrits des Naturalistes, des Medecins, et de l'Observation. Par M. *Amoreux*, fils, Docteur en Medecine en l'Université de Montpellier, Bibliothecaire, de plusieurs Academies et Sociétés d'Agriculture. 8vo. Paris, 1789.

### INDEX.

### A.

Page

ABILGAARD, F. L. de Inſtit. Veterin. Hafn. 431

Accademie delle Scienze di Napoli, Atti della, 111

Agnelli, Nic. de pœna funis, — — 223

Aiton, William, Hortus Kewenſis, — — 427

Alyon, M. Cours de Botanique, — — 219

Amoreux, M. des Inſectes venimeux, — — 433

Ancients, their obſcure Accounts of ſome Diſeaſes, — 371

Anderſon, Dr. J. on the Tanjore Antidotes for venomous Bites, — — — 285, 289

——, James, his Account of a Monſter, — 424

Arnemann, Juſtus, de Aphthis, — — 103

Arnica Montana, its medicinal Effects, — 236

Audirac, J. J. de Calore Animali, — — 431

### B.

Baillie, Dr. M. Caſe of tranſpoſed Viſcera, — 177

——, on a Change of Structure in the Ovarium, 322

Bang, F. L. Pharmacopoeia Noſoc. Frid. Hafn. — 223

Bark, Accounts of a new Species of, — 154, 158

Bartholin, Tho. his Deſcription of a Monſter, — 425

Barton, Ja. de Dyſenteria, — — 103

Baumes, M. ſur une Maladie du Meſentere, — 111

—— l'Ictere des Enfans, — — 224

Belleval, P. Richer de, Eloge de, — — — 219

Belloſte, M. his recommendation of a Decoction of Walnut Leaves in Ulcers, — — — 321

Bengal, Tranſactions of a Society in, — — 333

Berkenhout, Dr. J. Synopſis of Natural Hiſtory, — 213

Berlinghieri, L. V. della Theoria del Crawford, — 106

Bertrandi, Amb. Opere Anatomiche, — — *ibid.*

Bicchierai, A. dei Bagni di Montecatani, — 111

Bindon, Nic. de Ictero, — — 101

Births, numerous, Obſervations and Facts relative to, 83

Bladder, Effects of Mercury in a ſpaſmodic Affection of, 356

Bleeding, Remarks relative to, — — 139

Blumenbach, J. F. Inſtitut. Phyſiolog. — 106

—— Specimen Phyſiol. compar. — *ibid.*

Boehmer, G. R. de Cyano ſegetum, — — 215

Bormes, le Baron de, Reponſe a M. de Fourcroy, — 224

Breton, M. le, ſur les Eaux de Cheltenham, — 218

Buniva, M. F. Diſput. Phyſ. et Med. — — 108

### C.

Calculus, in the pulmonary Artery, Inſtance of, — 347

Callifen, H. Principia Chirurgiæ, — — 216

Camper, P. on the Operation of Lithotomy, —— 162

Cancer, of the Breaſt, Caſe of, — — 40

——————, Remarks on, —— — 53

Carminati, B. Opuſcula Therapeutica, — — 214

Carolis, D. F. de, Riſpoſta del, — — 106

Carter, Dr. F. Account of various medical Syſtems, — 428

Cataract, Remarks on, — — — 395

Cattle, compendious Syſtem of the Diſeaſes of, — 429

Chemant, M. de, ſur les Dents artificiels, — 218

Cheſham, epidemic Sore Throat at, deſcribed, — 7

Chriſtie, Thomas, Obſervations on Pemphigus, — 361

Clark, J. on Diſeaſes of Horſes, —— —— 211

Clowes, Tho. Caſe of Hernia, — — 72

Contagion, febrile, Remarks on, — — 260

Cortex Anguſturæ, Accounts of, —— 154, 158

Crane, J. on the Nottington Water, — — 428

Crichton, Dr. A. on the Lichen Iſlandicus & Arnica M. 229

Cryſtalline Humour, Method of extracting it, — 416

Cullen, Dr. W. on the Materia Medica, — — 211

Currie, Dr. his Experience of the Uſe of Wine in Tetanus, 299

Cyrilli, Dom. Plant. rar. Neapolit. — — 336

### D.

Darwin, Dr. Inſtance of Hydrophobia from Sympathy, 300

Davidſon, Tho. Facts relative to Small Pox, — 353

Dazille, M. ſur le Tetanos, — —— 104

Deglutition, impeded, Caſes of, —— — 356

Denman, Dr. Thomas, Introduction to Midwifery, 110

Detharding, G. G. de Utero inverſo, — — 430

——————— Cruſta inflammatoria, — *ibid.*

Domingue, St. Maladies Epizootiques de, — 111

Dorthés, M. Eloge de P. Richer de Belleval, —— 219

Douglas Dr. And. on the Rupture of the Uterus, — 100

Dropſy, Caſes of cured by blue Vitriol, — 149

Duffin, Mr. his Account of the Tanjore Snake Pill, 287

Durand, M, ſur les Fievres intermittentes, — 104

### E.

Eiſton, Joannes, de Ophthalmia, — — 102

Eſſays Medical, — — — — 429

Evans, G. J. de Dyſpepſia, — —— 102

Ewer, Dr. J. Account of a new Species of Bark, — 154

Eyerel, Joſ. Diſſert. de Morbis chronicis, — 216

F.

Fever, Epidemic, Account of, — — 117
Fevers, Treatife on, — — — 428
Forbes, A. G. de Incubo, — — — 102
Formey, L. de Syftem. abforb. patholog. — 108
Fortis, Abb. del Nitro Minerale, — — 105
Fothergill, Dr. John, French Tranfl. of his Obf. on the Ceffation of the Menfes, — — 224
Franco, P. his Method of cutting for the Stone defcribed, 166
Fuchs, G. F. Gefchichte des Zinks, — — 110

G.

Gaertner, Jof. de Fruct. et Semin. Plantarum, — 214
Garnet, Tho. de Vifu, — — — 102
Garthfhore, Dr. M. on numerous Births, — 83
Gaftric juice, recommended in the Bite of a rabid Animal, 306
Genettes, R. N. du Friche des, de Vafis lymphaticis, 432
Giornale fcientifico, — — — 223
Girtanner, C. ueber die venerifche krankheit, — 336
Gleditch, J. G. vermifchte botanifche abhandlungen, 223
Gmelin, J. F. Grundritz der Chemie, — — *ibid.*
Gregory, Dr. John, his Works, — — 335
Grieve, Dr. J. his Account of Koumifs, — — 197
Grimfton, J. Cafe of a fractured Scull, — 277
Guillemeau, J. L. de Signis virginitatis, — 215
Gunzius, J. G. de Cortice Salicis, — — 216

H.

Halloran, Silvefter O', Obfervations on Cataract, 395
Harper, A. Oeconomy of Health, — — 100
———— on Infanity, — — — 101
Harrington, Dr. R. Letter to Dr. Prieftley, — 212
Hartel, A. F. G. de Febre phthificorum, — 433
Haygarth, Dr. his Propofal for the Prevention of Hydrophobia, 295
Heart, remarkable Difeafe of, — — 341
Henderfon, Dr. W. on the Plague, — — 213
Hendy, Dr. J. Vindication of his Treatife on the Glandular Difeafe, — — — — 212
Hepatitis, Cafe of, — — — 142
Hermann, J. Amphibiorum virtutis medicatæ defenfio, 335
Hernia, Cafe of, — — — 72
Higgins, W. Comparative View of the phlog. and antiphlog. Theories, — — — 213
Hildebrandt, G. F. ueber die Pocken, — — 110
Hoffmann, G. F. Vegetabilia cryptogamica, — 107
Hopfon, Dr. C. R. General Syftem of Chemiftry, — 213
Howard, John, Account of Lazarettos, — 332
Hughes, T. Cafe of a Cancer of the Breaft, — 40

Humpage, Benj. on the Hydrocele, —— — 99

Hunckzowfky, J. ueber die Gefchichte der Chirurgie, 104

——————, on the Efficacy of a Decoction of green Walnut Shells in Ulcers, —— —— 319

Hydatids, Defcription of, —— — —— 77

Hydrophobia, Obfervations relative to, — 285, 291, 295

——————, from Sympathy, Inftance of, —— 300

J.

Jamefon, Thomas, on Diluents, — —— 100

Janfen, W. X. de Pelagra, —— —— 107

Impregnation, Speculations relative to, — — 428

Inoculation, Facts relative to, — — 353

Joerdens, J. H. Defcriptio nervi Ifchiadici, — 222

Iris, its Structure explained, —— —— 400

Juffieu, Ant. Laur. de, Genera Plantarum, — 430

K.

Keck, J. F. Abhandlungen aus der Arzneywiffenfchaft, 221
Kentifh, Dr. Advice to gouty Perfons, —— 334
Kinglake, R. Cafe of a remarkable Difeafe of the Heart, 341
Kirwan, R. German Tranflation of his Works, — 104
——————, Effay on Phlogifton, — — 333
——————, ——————French Tranflation of, 111
Kite, C. on the Recovery of the apparently Dead, 99
Kittfon, Geo. Rich. de Febribus, — — 102
Knigge, T. Medicinifche fragmente, — — 221
Koumifs, Account of its medicinal properties, — 197
Kraufe, C. C. Opufcula Academica, — — 107
——————, K. C. von dem einfluffe der Einbildungfkraft, 221
Kulmus, J. A. Anatomifche Tabellen, — — 433
Kumpel, J. A. F. de Magnetifmo, — — 431

L.

Lavoifier, M. Traité de Chimie, — — 219
Lawfon, G. M'Farquhar, de Calculo Urinario, — 102
Leeds, Refult of two general Inoculations at, — 270
Lettfom, Dr. J. C. on the Effects of hard Drinking, 335
Lichen Iflandicus, Obfervations on its Effects, — 229
Lind, Dr. J. on the Tænia Hydatigena, —— 76
Link, H. F. de analyfi Urinæ, — — 431
Lithotomy, Operation for, at two different Times, defcribed, 162
London Medical Journal, Flemifh Tranflation of, — 433
Lucas, James, on febrile Contagion, — — 260

M.

Machy, M. de, Manuel du Pharmacien, —— 112

Maclaurin, J. C. de fluxu menftruo, — — 102

Macquart,

Macquart, M. Memoires de Mineralogie, —— 218

Madras, Account of the Weather at, — — 210

Maincourt, L. F. de Polypis, — — 222

Martino, Padre de S. ſopra la fermentazione, — 105

Maſcagni, P. de Vaſis Lymphaticis, — — 214

May, Dr. W. Account of an Epidemic Fever, — 117

Medical Society in London, Memoirs of, — 334

Meiners, C. ueber den thieriſchen Magnetiſmus, — 110

Mercury, its Effects in impeded Deglutition, —— 356

———, ——— Spaſm of the Bladder, —— 360

Mithoff, Gul. Diſſert. Medico-Obſtetricia, — 432

Moeller, J. de Medicam. Antimon., — — 222

Monro, Dr. D. Appendix to his Treatiſe on Chemiſtry, 212

Monſter, Deſcription of, — — — 422

Montecatani, dei bagni di, — — — 111

Montpellier, Aſſemblée publique de la Soc. Roy. de, 220

Moore, James, on the healing of Wounds, — 427

Morell, C. F. ueber der Geſundbrunnen der Schweitz, 220

Moſcheni, S. dell' aria Inflammabile, — — 110

Moſſman, Dr. G. on the Brunonian Practice of Phyſic, 334

Murray, A. J. G. de redintegratione partium, — 108

———, J. A. de Magnetiſmo Animali, —— 432

N.

Neubeck, V. G. de Natatione frigida, — — 431
Nicolas, M. Leçons de Chimie, — — 103

O.

Ovarium, particular Change of Structure in, —— 322

P.

Paré, Ambroſe, referred to on the ſubject of tranſplanting Teeth, 243

———, his Deſcription of a Monſter, —— 426

Paris, Hiſtoire de la Societe Royale de Medecine, — 220

Paſta, G. dell' opio nelle Malattie veneree, —— 217

Patten, S. Caſes of impeded Deglutition, —— 356

Pemphigus, Obſervations on, — — 361

———, not diſtinctly deſcribed by the Ancients, 370

———, different Species of, — — 372

———, Treatment of, —— —— 392

Percival, Dr. T. on the Rabies Canina, — — 295

Pliſſon, M. ſur un moyen de guerir les douleurs de Dents, 105

Prevoſt, M. des Forces magnetiques, — — 112

R.

Rabies Canina, ſee Hydrophobia, — —

Rahn, J. H. de Sympathia, — — — 223

Ramſay, Joannes, de alimento Hominum, — 102

Rattle-

Rattle-fnake, Facts relative to its Poifon, — 310, 312
Reeve, Thomas, on Eryfipelatous fore Throat, — 430
Reichel, Baron, his Account of a Monfter, — 422
Reufs, C. F. Difpenfatorii fupplementum, — 107
Riegels, N. de fatis Chirurgiæ, — — 108
Rowley, Dr. W. on female Difeafes, — — 211
Rumfey, Henry, Account of an Epidemic fore Throat, 7
Rufh, Dr. Benjamin, medical Enquiries, — 427

S.

Sartius, M. de Conftit. epidem. Cafulan., — 109
Schlegel, J. C. Thefaurus Path. Therap., — 215
——————, Sylloge opufc. de Sympathia, — 216
Schneiter, J. G. Amphibiorum virtutis medicatæ defenfio, 335
Schulze, J. E. F. de Toxicologia veterum, — 431
Schweighæufer, J. F. Amphibiorum virtutis medicatæ defenfio, 335
Scull, Cafe of Fracture of, unfuccefsfully treated, — 277
Scuderi, F. M. del Vajuolo, — — — 217
Sheldon, John, on the Fracture of the Patella, — 101
Sherlock, Jof. de exercitatione, — — 103
Siebold, G. C. de effectibus opii, — — 432
Skin, Engravings, to illuftrate its Difeafes, recommended, 366
Small Pox, Means ufed to prevent it from fpreading, 263
—————, Facts relative to, — — — 353
Smith, Dr. J. French Tranflation of his Work on Cheltenham Water, — — — 218
—————, J. E. Icones Plantarum, — — 426
Sommer, J. C. ueber einer Kayfergeburt, — 222
Spedicati, D. ueber den Scharbock, — — 109
Spence, Geo. on the Difeafe confequent to tranfplanting Teeth, 243
Steyrers, J. P. Handbuch der Apothekerkunft, — 221
Stoll, Maxim. de Morbis chronicis, — — 215
Strack, C. von der allgem. krankenhaus in Mainz, — 336
Swartz, Olof. nova genera plantarum, — — 107
Sydenham, Dr. Tho. new Edition of his Works, — 99
Synochus, confidered as a variety of Typhus, — 125

T.

Tænia, hydatigena, defcribed, — — — 77
Tanjore Antidotes for venomous Bites, Account of, — 283
Taylor, B. on the Atmofphere of London, — 334
Teeth, Obfervations on a Difeafe confequent to the tranfplanting of, — — — 243
Tetanos, in many Particulars faid to refemble Hydrophobia, 299
————, good Effects of Wine in a Cafe of, — *ibid.*
Tibia, Inftance of the Application of the Trepan to it, 80
Tobacco, Treatife on, — — — 420
Toggia, F. delle Malattie del Cavallo, — 222
Toulmin, Dr. G. H. Inftruments of Medicine, — 334

Tranfplanting of Teeth, Obfervations on a Difeafe confequent to, — — — 243
Trepan, Account of its Application to the Tibia, — 80
Trotter, Tho. de Ebrietate, — — 103
Turin, Memoires de l'Academie des Sciences, — 433

V.

Vandelli, Dom. Viridarium Grifley Lufitanicum, — 335
Venæfection, numerous Inftances of, in one Patient, 345
Verguin, M. Cafe of a difeafed Tibia, — 80
Vifcera, tranfpofed, Cafe of, — — 178
Vitriol, Blue, efficacy of in Dropfy, — 149
Underwood, Dr. M. on Difeafes of Children, — 333

W.

Wallis, Dr. G. his Edition of Dr. Sydenham's Works, 99
Walnuts, a Decoction of the Green outer Shell of, recommended in Ulcers, — — — 319
Werner, P. C. de vermibus inteft., — — 108
Wilkinfon, G. Cafe of Hepatitis, — — 142
Willdenow, C. L. de Achilleis, — — 215
Williams, Dr. A. Account of a new Species of Bark, 158
Wilfon, Joannes, de Diureticis, — — 103
Witting, C. F. de Tartaro Emetico, — 335
Wright, Dr. W. on the efficacy of Blue Vitriol in Dropfy, 149

Z.

Zandycke, F. A. van, Flemifh Tranflation of the London Medical Journal, — — — 433
Zehner, J. G. de febre puerperarum, — 215
Zwierlein, K. A. vermifchte fchriften, — 221

